# Protective Role of *γδ* T Cells in Different Pathogen Infections and Its Potential Clinical Application

**DOI:** 10.1155/2018/5081634

**Published:** 2018-07-10

**Authors:** Yueshui Zhao, Ling Lin, Zhangang Xiao, Mingxing Li, Xu Wu, Wanping Li, Xiaobing Li, Qijie Zhao, Yuanlin Wu, Hanyu Zhang, Jianhua Yin, Lingling Zhang, Chi Hin Cho, Jing Shen

**Affiliations:** ^1^Laboratory of Molecular Pharmacology, Department of Pharmacology, School of Pharmacy, Southwest Medical University, Luzhou, Sichuan, China; ^2^School of Biomedical Sciences, Faculty of Medicine, The Chinese University of Hong Kong, Shatin, Hong Kong

## Abstract

*γδ* T cells, a subgroup of T cells based on the *γδ* TCR, when compared with conventional T cells (*αβ* T cells), make up a very small proportion of T cells. However, its various subgroups are widely distributed in different parts of the human body and are attractive effectors for infectious disease immunity. *γδ* T cells are activated and expanded by nonpeptidic antigens (P-Ags), major histocompatibility complex (MHC) molecules, and lipids which are associated with different kinds of pathogen infections. Activation and proliferation of *γδ* T cells play a significant role in diverse infectious diseases induced by viruses, bacteria, and parasites and exert their potential effector function to effectively eliminate infection. It is well known that many types of infectious diseases are detrimental to human life and health and give rise to high incidence of illnesses and death rate all over the world. To date, there is no comprehensive understanding of the correlation between *γδ* T cells and infectious diseases. In this review, we will focus on the various subgroups of *γδ* T cells (mainly V*δ*1 T cells and V*δ*2 T cells) which can induce multiple immune responses or effective functions to fight against common pathogen infections, such as *Mycobacterium tuberculosis*, *Listeria monocytogenes*, influenza viruses, HIV, EBV, and HBV. Hopefully, the gamma-delta T cell study will provide a novel effective way to treat infectious diseases.

## 1. Introduction

Infectious diseases are mainly caused by pathogen infection (including viruses, bacteria, and parasites). Many types of infectious diseases are detrimental to human life and health and give rise to high incidence of illnesses and death rate all over the world [1]. Dual infection by different types of viruses and a secondary infection is a common clinical phenomenon, which threatens the health of human beings [2–4]. At the beginning, major focus has been put on pathogens instead of host immune response [5]. But pathogens develop chemical resistance which causes a decrease in curative effect [6, 7]. Therefore, more and more researchers are focusing on conventional T cells and their subpopulations with different phenotypes [8–11]. However, the study on the function and immune response of unconventional T cells (*γδ* T cells) is still neither enough nor systematic. In this review, we will introduce the direct and indirect effector function and immunity of *γδ* T cells in detail in a variety of pathogen infections in the hope to provide more information for clinical treatment based on the better understanding of the function of different subsets of gamma-delta T cells.

*γδ* T cells, a subgroup of T cells based on the different T cell receptor (TCR), when compared with conventional T cells (*αβ* T cells), make up a very small proportion of T cells. They are widely distributed in different parts of the human body [12]. *γδ* T cells are mainly divided into three subgroups according to the expression of *γ* (including 2/3/4/5/8/9) and *δ* (including 1/2/3/5) chains: V*δ*1 T cells, V*δ*2 T cells, and V*δ*3 T cells [13]. Specifically, V*δ*1 gene is paired with different V*γ* elements (including V*γ*2/3/4/5/8), V*δ*2 gene is paired with V*γ*9 chain, and V*δ*3 gene is associated with V*γ*2 or V*γ*3 [14]. The distribution and function of different subgroups of *γδ* T cells are strikingly different.

V*δ*1 T cells are mostly found in the mucosal epithelium and are in connection with infection of many pathogens [15], such as *Listeria monocytogenes*, human immunodeficiency virus (HIV), and cytomegalovirus (CMV) [16–21]. V*δ*2 T cells are primarily enriched in circulating blood. V*δ*2 T cells are uniquely matched with V*γ* gene usage of V*γ*9 (termed V*γ*9V*δ*2) and they make up the majority of *γδ* T cells in the peripheral blood [22, 23]. V*δ*2 T cells also exhibit their effective immune response to bacteria and viruses (like mycobacteria, influenza viruses, and Epstein–Barr virus) like V*δ*1 T cells [24–27]. V*δ*2 T cells based on expressing CD27 and CD45RA are segmented into four different functional subsets with respective characteristic: CD45RA^+^CD27^+^ (naïve), CD45RA^−^CD27^+^ (central memory without effector function which are rich in lymph nodes), CD45RA^−^CD27^−^ (effector memory), and CD45RA^+^CD27^−^ (terminal differentiation which massively appears in the inflammatory site) [28, 29]. They play a significant role via their effector functions and memory responses during infections [28]. The natural killer cell receptor (NKG2D) and Toll-like receptors (TLRs) are also expressed on the surface of both V*δ*1 T cells and V*δ*2 T cells to exert their effector function during infections even in tumor immunity [30–32]. In contrast with V*δ*1 T cells and V*δ*2 T cells, V*δ*3 T cells, the smallest subset of *γδ* T cells, are abundant in the liver and are mainly involved in the process of chronic viral infections [33, 34].

In addition, *γδ* T cells are categorized into a suite of multiple functional populations as follows: IFN-*γ*-producing *γδ* T cells, IL-17A-producing *γδ* T cells, and antigen-presenting *γδ* T cells. They indirectly promote immune response against pathogen infection by *γδ* T cells themselves or other immune cells (like CD8^+^ T cell and B cells) [35–37].

Murine *γδ* T cells also have various subsets on the basis of characteristic V*γ* usage (including1/2/3/4/5/6/7): V*γ*1 combined with V*δ*6.3, V*γ*5 paired with V*δ*1, V*γ*6 paired with V*δ*1, and V*γ*7 paired with three diverse V*δ* elements (including V*δ*4/5/6) [38]. Interestingly, human V*δ*1 cells are the primary subtypes found at mucosal surfaces and share certain characteristics with murine *γδ* intraepithelial lymphocytes (which are associated with V*γ*7) [39]. On the contrary, V*γ*9V*δ*2 T cells are restricted to certain species and are found only in humans and higher primates [39].

## 2. *γδ* T Cells Recognize Antigens

*αβ* T cells which depend on antigen presentation and restrictive major histocompatibility complex (MHC) molecules recognize antigens. *γδ* T cells, however, can recognize various types of antigens (including nonpeptide antigens and stress-induced ligands) without restrictive MHC molecules [40]. Mounting evidence indicates that *γδ* T cells exert their protective function in elimination of pathogens and tissue repair via producing cytokines, chemokines, and lytic enzymes, cytotoxic and noncytolytic antiviral activities, and so on [41].

Based on the diverse subtypes, *γδ* T cells could recognize different types of antigens. V*δ*1 T cells could recognize MHC class I chain-related antigens A and B (MICA/B) and stress-induced molecules frequently expressed on epithelial cell in a *γδ* TCR-dependent manner [40, 42–44]. Activated V*δ*1 T cells could exert their effector function against bacterial infection and kill infected cells by production of interleukins and interferons [45]. Interestingly, MICA/B expressed on cancer cell are recognized by both V*δ*1 T cells and V*δ*2 T cells but in a NKG2D-dependent manner [46, 47]. In addition, V*δ*1 T cells respond to MICA-related UL16-binding proteins (ULBPs) based on their ability to combine with human cytomegalovirus (HCMV) glycoprotein UL16 in the same manner [48, 49]. ULBPs are a family of MHC class I-related human cell surface molecules and ligands of NKG2D which play a key role in regulation of innate and adaptive immune responses [50, 51]. Lipids and glycolipid which are relevant to various bacteria (like mycobacteria) are required for the presentation of MHC-related class Ib molecules which are expressed on antigen-presenting cells (APCs), and thus, the bacteria-derived antigens can be recognized by V*δ*1 T cells [52–55].

**Figure 1:**
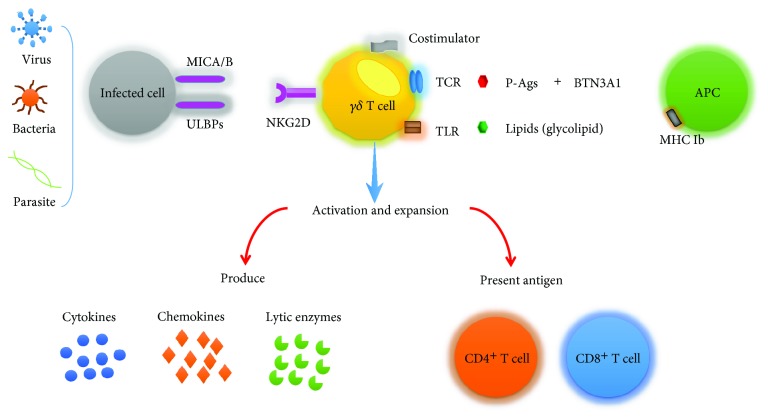
*γδ* T cells recognize antigens. Diverse subtypes of *γδ* T cells could recognize different types of antigens. *γδ* T cells (both V*δ*1 and V*δ*2) could recognize stress-induced molecules MICA/B and ULBPs which are expressed in cancer and transformed and infected cells in a NKG2D-dependent manner. V*δ*1 T cells could recognize bacteria-derived antigens (including lipids and glycolipid) via MHC-related class Ib molecules which are expressed on antigen-presenting cells. V*δ*2 T cells recognize phosphoantigens via forming tight complexes following binding with BTN3A1, and in the context of costimulators, V*δ*2 T cells are activated and expanded. V*δ*3 T cells can be activated by CD1d which may combine with glycolipid and kill CD1d target cells. Activated V*δ*2 T cells and V*δ*1 T cells could activate the expression of Toll-like receptors which have the capacity to recognize infectious pathogen-associated molecule patterns. Activation and proliferation of *γδ* T cells exert their potential effector functions via producing cytokines, chemokines, and lytic enzymes, performing cytotoxic and noncytolytic antiviral activities, presenting antigens to CD4^+^ and CD8^+^ T cells, inducing maturation of dendritic cells (DCs), providing B cell help, and so on.

V*δ*2 T cells, in particular, are activated by low molecular weight nonpeptidic antigens (also called phosphoantigens (P-Ags)) which are produced by transformed cells or cells infected by pathogens (such as viruses and bacteria) [56, 57]. IPP (isopentenyl pyrophosphate) and HMBPP ((E)-4-hydroxy-3-methyl-but-2-enyl pyrophosphate) are the most prominent ones. In general, P-Ags associated with infected or transformed cells are produced by way of the mevalonate pathway (like IPP) when compared with the microbes in the isoprenoid pathway (like HMBPP) [58, 59]. In other words, P-Ags generated by diverse cells and different metabolic pathways are different to each other. For example, HMBPP primarily comes from *Mycobacterium tuberculosis*, *Listeria monocytogenes*, and so on [57]. Some clinical medicines can alter the intracellular level of P-Ags to some degree. Nitrogen-containing bisphosphonates (N-BPs) and statins (a kind of anticholesterol drugs) are the most common medicines to increase or decrease the P-Ag level via inhibiting the P-Ag-relevant enzyme [60]. The level of P-Ags also has an obvious trend of increase during stress and infection. Antigen presentation to V*δ*2 T cells is independent of restrictions of MHC molecules [60]. Early studies suggested that V*δ*2 T cells recognize P-Ags by presentation of CD1d which is expressed on APCs (such as dendritic cells) and monocytes [52, 61]. Butyrophilin 3A1 (BTN3A1) is involved in the process of presenting P-Ags [62]. BTN3A1 binds with P-Ags by its B30.2 domain, and finally, P-Ags are recognized by V*δ*2 TCR [63, 64]. Besides, BTN3A1 combined with P-Ags also plays an import role in the process of activation of V*δ*2 T cells following N-BP treatment [63]. Like *αβ* T cells, the activation and proliferation of V*δ*2 T cell also need the second signals which depend on costimulators including CD40-CD40L, CD28-B7.1/7.2, CD137 (4-1BB), and CD2 [65, 66]. Toll-like receptors, as the most common pathogen recognition receptors, have the capacity to recognize infectious pathogen-associated molecule patterns [32]. Activated V*δ*2 T cells and V*δ*1 T cells could activate the expression of Toll-like receptors in reverse [32]. After activation, V*δ*2 T cells exert their potential effector functions in the following ways: producing cytokines, chemokines, and lytic enzymes; performing cytotoxic and noncytolytic antiviral activities; inducing maturation of dendritic cells (DCs); providing B cell help; and presenting antigens to CD4^+^ and CD8^+^ T cells (Figure 1).

V*δ*3 T cells can be activated by CD1d which may combine with glycolipid and kill CD1d target cells and release different kinds of cytokines (includingTh1, Th2, and Th17) and even promote maturation of DC into APCs [33].

## 3. Function of *γδ* T Cells in Infectious Diseases

In early report, researchers pay more attention on *αβ* T cells' protective immunity during infectious diseases. But there is no systematic understanding on *γδ* T cells' direct or indirect protective ability to fight against pathogens. This review will summarize the diverse functions of *γδ* T cells in various infectious diseases.

### 3.1. Bacteria

#### 3.1.1. *Mycobacterium tuberculosis* (MTB)

*γδ* T cells play a significant role in MTB infection. Interestingly, V*γ*9V*δ*2 T cells which exist in humans and the vast majority of nonhuman primates carry huge weight in mycobacterial infections [67]. On the contrary, V*δ*1 T cells seem to be more relevant to other infectious diseases, such as HIV diseases [68].

V*γ*9V*δ*2 T cells recognize HMBPP via forming tight complexes following binding with BTN3A1 during MTB infection. In the presence of costimulators, V*γ*9V*δ*2 T cells are subsequently activated and expanded [69]. Recently, a number of studies show that phosphoantigen HMBPP and many cytokines participate not only in expansion but also in recall-like expansion and effector functions of V*γ*9V*δ*2 T cells after MTB infection [24]. Compared with CD4^+^ T cells, HMBPP-activated V*γ*9V*δ*2 T cells produce a speck of IL-2 which contributes to the proliferation of unconventional T cells. It has been demonstrated in cynomolgus monkeys that low-dose IL-2 could synergize with nitrogen-containing bisphosphonate or pyrophosphomonoester drugs to expand V*γ*9V*δ*2 T cells [70]. Similarly, in nonhuman primate models, HMBPP together with IL-2 maximizes its stimulating effect [71]. Besides, T cell growth cytokines (like IL-15 and IL-21) and Th17-related cytokines are also involved in the above process [24]. After V*γ*9V*δ*2 T cells are activated and proliferated, they take part in the process to fight against MTB. In early years, Gercken et al. [72] have already proven that the mononuclear phagocytes as accessory cells infected by MTB could activate *γδ* T cells and rest upon costimulators to show a number of functions, especially secretion of cytokine and expression of cytolytic effectors. Generally, MTB phosphoantigen-activated *γδ* T cell produces TNF-*α* and IFN-*γ* to enhance the protective responses to MTB [73]. Meanwhile, cytolytic effector function based on granulysin and perforin is essential for *γδ* T cell to defend against the MTB infections. There is direct evidence that *γδ* T cell inhibits and even kills the intracellular MTB by granulysin and perforin with bactericidal ability in macaque models [74]. In addition to the above anti-MTB effects of *γδ* T cell, it is newly discovered that activated *γδ* T cell may stimulate the maturation of DCs to modulate other cells (like CD4 T helper cells and B cells) to enhance immune response to MTB [75–77]. Phenotype differentiation of V*γ*9V*δ*2 T cells also help to strengthen the effective function of *αβ* T cells to fight against MTB, like promoting CD4^+^ and CD8^+^*αβ* T cells to secrete TNF-*α* and IFN-*γ* to kill MTB [78]. Research evidence also suggests that memory response of V*γ*9V*δ*2 T cells may be based on its phenotype differentiation, and further research is needed to unveil the exact mechanism [25].

Overall, the immune response of HMBPP-activated V*γ*9V*δ*2 T cells to fight against MTB is dependent on secretion of cytokine, expression of cytolytic effector function, and maturation of DCs.

#### 3.1.2. *Listeria monocytogenes*

*Listeria monocytogenes (L. monocytogenes)* is an intracellular bacterium and exists in food (like meat and other dairy products). It can cause a wide range of foodborne diseases in both animals and human [79]. *L. monocytogenes* can cross the blood-brain barrier, intestinal barrier, or feto-placental barrier and lead to serious infectious illness and death in different populations [80].

IL-17A is mainly produced by *γδ* T cells during *L. monocytogenes* infection to promote innate and adaptive immune responses, and it promotes host function of effective elimination of infection by producing cytokines and CXC chemokines [81–84]. Herein, the proliferation and accumulation of neutrophils depending on cytokines and CXC chemokines induced by IL-17A are involved in cross-priming to CD8^+^ T cells during *L. monocytogenes* infection [85]. In the early infective stage in the liver of mouse models, IL-17A produced by *γδ* T cells enhances the antibacterial activity of nonphagocytic cells infected by *L. monocytogenes*, which is involved in promoting antimicrobial peptide mouse *β*-defensin (mBD) gene expression [86]. Besides, the IL-17A-producing *γδ* T cells which are activated rapidly following *L. monocytogenes* infection mediate its antibacterial immune response via IL-23 production by pathogen-activated macrophages/DCs during the early phase of infection [86]. Moreover, in the IL-17A^−/−^ mouse model, following *L. monocytogenes* infection, the bacterial burden in the spleen and liver was significantly higher than that of control mice within the stipulated time [87, 88]. Therefore, it can be concluded that IL-17A plays a significant role in the innate immune response to *L. monocytogenes*. Subsequently, IL-17A has been proven to be indispensable in cytotoxic T cell response against primary *L. monocytogenes* infection. It can also promote the expansion of cytotoxic T cell (CD8^+^ T cell). Collectively, innate IL-17A produced mainly by *γδ* T cells could induce the proliferation of cytotoxic T cell and play their effective cytotoxic T cell response to eliminate *L. monocytogenes* [87, 88].

IL-17A also plays a crucial role in controlling intestinal pathogens during secondary *L. monocytogenes* infection. In the mouse model infected with the internalin A mutant recombinant strain of *L. monocytogenes* (which simulate human intestinal invasion conditions), V*γ*4^+^ memory *γδ* T cells are confirmed as resident memory (Trm) population in the mesenteric lymph nodes (MLNs) [18]. *γδ* Trms exert effective elimination of bacteria by early IL-17A secretion to mediate the process in which *γδ* Trms contain the bacteria within granulomas in the liver and form large clusters with myeloid cells (including neutrophil) at the sites of *L. monocytogenes* replication foci [18].

### 3.2. Viruses

#### 3.2.1. Influenza Virus

Due to annual cocirculation and rapid spreading, influenza viruses lead to a large amount of global morbidity and mortality. Influenza viruses widely spread not only from children to the elderly but also to the diverse crowds [89]. Influenza viruses could be divided into the following categories: influenza A viruses (IVA), influenza B viruses (IVB), and influenza C viruses (IVC). IVA show a much more severe infection when compared with IVB and IVC viruses. IVA are derived from swine and avian species and can infect the human respiratory tract through several ways of virus transmission. Recently, researchers are increasingly focusing on the establishment of mouse models following avian influenza H5N1 infection to explore a nicely controlled mechanism of influenza virus infection by gamma-delta T cells [90].

Innate immunity acts as a frontline defense to eliminate virus by interferon and at the same time enhance the adaptive immune response [91]. Phosphoantigen-activated *γδ* T cells secret substances associated with killing cells infected by influenza viruses to fight against viruses, such as perforin, granzyme B, and granulysin [92, 93]. In humanized mouse models, phosphoantigen treatment significantly decreased weight loss and mortality associated with IVA infection and could control human IVA infection possibly via the selective activation and expansion of human V*δ*2 T cells. Thus, phosphoantigen-activated *γδ* T cells have a significant ability to clear human and avian influenza viruses [90]. In addition, *γδ* T cells also assist in strengthening the activity of APCs by providing significant signal molecules. After that, APCs play their antigen-presenting role to present influenza antigens to acquire T cells (like CD8^+^ T cells and CD4^+^ T cells) and influenza viruses will finally be cleared by these antigen-specific T cell responses. Moreover, phosphoantigen-activated and expanded *γδ* T cells also induce the expression of CCR1 [94]. CCRs are inflammatory chemokine receptors that promote the ability of elimination of viruses [92, 93].

The number of activated and proliferating *γδ* T cells, however, varies from person to person after influenza vaccination. Studies compared the number of activated and proliferating *γδ* T cells between young and elderly healthy human measured by flow cytometry following vaccination. It has been discovered that elderly individuals have lower number and slower kinetics changes of activated and proliferating *γδ* T cells than young men. It can be concluded from the study that age serves as an important factor to affect the efficiency of T cell response and may make vaccination have a severe drop-off in effectiveness [95].

Besides phosphoantigen and age, type I IFNs and other cytokines could also influence *γδ* T cell immune response against influenza infection [96, 97]. In the mouse model infected with IVA, researchers exposed IVA-infected mice to smoke or air. Mice exposed to chronic cigarette smoke recovered poorly from primary influenza A pneumonia but recruited *γδ* T cells to the lungs that predominantly expressed IL-17A. Depletion of IL-17A significantly increased T-bet expression in *γδ* T cells and improved recovery from acute IVA infection [97]. Collectively, cytokines and phosphoantigen play a crucial part in *γδ* T cell-mediated antiviral immune response during influenza virus infection.

#### 3.2.2. Human Immunodeficiency Virus (HIV)

**Figure 2:**
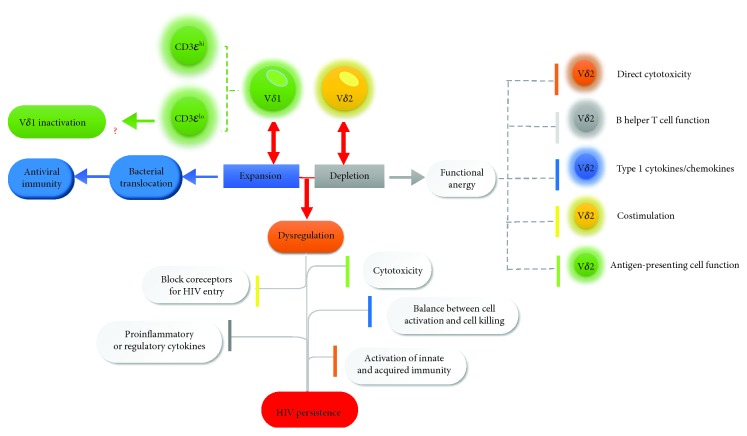
Dysregulation of *γδ* T cells during human immunodeficiency virus (HIV) infection. Expansion of V*δ*1 T cells during HIV infections was associated with microbial translocation which has relevance to immune activation and exhibited its antiviral immune response. Recently, V*δ*1 T cells are segmented into two subsets: CD3*ε*lo V*δ*1 T cells and CD3*ε*hi V*δ*1 T cells, and CD3*ε*lo V*δ*1 T cells may at least partially induce V*δ*1 T cell inactivation based on its lower responsiveness to antigenic stimulation. However, the number and function of V*δ*2 T cells are depleted during HIV infection. Depletion of V*δ*2 T cells leads to inefficient immune response to HIV with inhibited direct cytotoxicity, B helper T cell function, type 1 cytokine or chemokine secretion, antigen-presenting cell function, and costimulation of NK cells. The lopsided proportion of V*δ*1 and V*δ*2 T cells causes a negative response against HIV infection with inhibited cytotoxicity, coreceptor for HIV entry, proinflammatory or regulatory cytokine release, activation of innate and acquired immunity, and imbalance between cell activation and killing. Thus, dysfunction of *γδ* T cells leads to HIV immune evasion and finally causes chronic infection.

HIV infection is different from other viral infections that it does not depend on any one *γδ* T cell subset alone but need two primary subsets of *γδ* T cells to participate together [98]. The percentage of two subsets of *γδ* T cells, however, can be changed or reversed during HIV infections [99]. V*δ*1 and V*δ*2 T cells in good proportion would play a key role in HIV infections. It has been reported that increasing V*δ*1 during HIV infection correlated with the proliferation of CD8^+^ T cells [100]. Recently, researchers found that the changes in *γδ* T cell and CD8^+^ T cell in primary and chronic stages of HIV infection (PHI and CHI) are different. Specifically, in untreated chronic HIV infection (UT-CHI), researchers found a positive correlation between *γδ* T cell frequency and CD8^+^ T cell activation. In contrast, in primary HIV Infection (PHI) patients, a negative correlation was found [101]. In addition to V*δ*1 and CD8^+^ T cells, there is a correlation between V*δ*2 T cells and CD4^+^ T cell and they are inversely associated with viral loads [102]. Moreover, inversion of the V*δ*2-to-V*δ*1 ratio was detected before the inversion of the CD4-to-CD8 ratio, which suggests that the abnormal percentage of V*δ*1 and V*δ*2 T cells also affected the CD4^+^ to CD8^+^ T cell ratio [103]. Recent studies highlight that the CD4/CD8 ratio may serve as a better biomarker for assessing disease progression and HIV's immune suppression in HIV-infected population [104]. It is also supported by another finding that there is a significant relationship between early levels of soluble biomarkers and exhausted CD4/activated CD8 T cells via systematic analysis of correlation between soluble inflammatory biomarker expression and CD4/CD8 T cells at the different stages of HIV infection (including PHI, CHI, and UT-CHI) in HIV-infected Mozambican adults [105]. The lopsided proportion of V*δ*1 and V*δ*2 T cells causes a negative response against HIV with inhibited cytotoxicity of *γδ* T cells to kill HIV-infected cells, inhibited secretion of proinflammatory cytokines which is associated with antiviral ability, inhibited ability to block coreceptors for HIV entry, inhibited activation of innate and acquired immunity, and imbalance between cell activation and killing [106, 107]. Thus, dysfunction of *γδ* T cells leads to HIV immune evasion and finally causes chronic infection [98] (Figure 2). Recently, it was reported that in acute HIV-1 infection, the phenomenon of the lopsided proportion of V*δ*1 and V*δ*2 T cells can be reversed by syphilis coinfection.

The effects of both V*δ*1 and V*δ*2 T cells to defend against HIV have been identified in past years [19]. Expansion of V*δ*1 T cells was associated with microbial translocation which has relevance to immune activation [108]. Recently, researchers found that HIV-infected patients have a higher percentage (but not absolute numbers) of V*δ*1 T cells [109]. Interestingly, according to the expression of the *ε* chain of the CD3 protein which is used for TCR signaling, V*δ*1 T cells can be segmented into two subsets: CD3*ε*^lo^ V*δ*1 T cells and CD3*ε*^hi^ V*δ*1 T cells [109]. CD3*ε*^lo^ and CD3*ε*^hi^ T cells have diverse phenotypes and functions. CD3*ε*^lo^ cells frequently express terminally differentiated (TD) cells, exhausted phenotypes, and programmed death-1 (PD-1) and fail to produce IL-17, suggesting that CD3*ε*^lo^ V*δ*1 T cells have a lower responsiveness to antigenic stimulation than CD3*ε*^hi^ V*δ*1 T cells [109]. This study indicates that HIV may partially induce V*δ*1 T cell inactivation and inhibit their effector functions to control virus during HIV infection. V*δ*2 T cells exhibited their functions in multiple ways when compared with V*δ*1 T cells. Phosphoantigen-activated V*δ*2 T cells have direct cytotoxicity for HIV-infected cells even for tumor cells and exhibit B helper T cell function [110–112]. Besides, activated V*δ*2 T cells have immune response by producing type 1 cytokines or chemokines including IFN-*γ*, TNF-*α*, RANTS, and MIP [106, 113, 114]. In the context of diverse kinds of chemokines (especially *β*-chemokine), V*δ*2 T cells can inhibit coreceptors for HIV entry [110, 115]. V*δ*2 T cells, in addition to being immune cells, are also confirmed as APCs [116]. Interestingly, antigen-stimulated *γδ* T cells costimulate NK cells and increase NK cell killing of autologous DC (editing) which is impaired in HIV^+^ patients [117]. Interaction between DC and *γδ* T cells also plays a key role in immune response to pathogen infections and virus-induced immune evasion [118]. Especially, in HIV-1 infection, exposure of DC to HIV-1 leads to its dysfunction but inversely stimulates *γδ* T cell proliferation and IFN-*γ* secretion via CCR5-mediated mechanism and plays a crucial role in controlling of HIV-1 replication, virus dissemination within DC via CCL4-mediated mechanism, and HIV-1 transfer to susceptible CD4^+^ T cells [119].

Effector function of V*δ*2 T cells and V*δ*1 T cells at different stages of HIV infection, namely, PHI and CHI, is remarkably different. V*δ*2 T cells are reported as potential regulatory T cells (Tregs) and play a crucial role in controlling immune activation by anti-inflammatory cytokine secretion during P-HIV [101]. Compared with C-HIV, both mucosal V*δ*2 T cells and V*δ*1 T cells exert more effective antiviral response in P-HIV [115].

Above all, V*δ*2 T cells act as a bridge between innate and acquired immunity to eliminate HIV. However, study shows that the number and function of V*δ*2 T cells are depleted during HIV infection [120]. Depletion of V*δ*2 T cells is caused by activation of the p38-caspase pathway via combination of HIV and CC chemokine receptor (CCR5) and integrin a4*β*7 [121]. There is no doubt that the depletion of V*δ*2 T cells leads to the inefficient immune response to HIV.

Though the majority of V*δ*2 T cells are decreased in HIV infection, activated CD16^+^ V*γ*9V*δ*2 T cells as a subset of V*γ*9V*δ*2 T cells (based on expression of Fc receptor for IgG, also called CD16) have the capacity to induce antibody-dependent cell-mediated cytotoxicity (ADCC) and exert their antiviral functions in HIV type 1 disease [122]. In an earlier report, V*δ*2 T cells expanded by zoledronate (one kind of bisphosphonates) and IL-2 are capable of enhancing ADCC cytotoxic effectors in HIV patients [107].

#### 3.2.3. Epstein–Barr Virus (EBV)

EBV, a virus related to transformation of B cell, could cause severe infections in individuals and more likely cause diseases including acute infectious mononucleosis, chronic active EBV infection, Burkitt lymphoma, and tumor (nasopharyngeal carcinoma) [123–125]. There were initial reports that cytotoxic lymphocytes have important influence on anti-EBV action, such as adaptive CD8^+^ T cell responses [126, 127]. Recently, it has been reported that innate cytotoxic lymphocyte participates in EBV infections [128]. NK cells and V*γ*9V*δ*2 T cells also exert their cytotoxic lymphocyte function against EBV infection [128]. Furthermore, latent EBV infection shows much a more significant increase in the expansion of both natural killer cells and V*γ*9V*δ*2 T cells when compared with lytic EBV infection [129]. Expanded V*γ*9V*δ*2 T cells interact with P-Ag which is produced by the mevalonate pathway by TCR of V*γ*9V*δ*2 T and BTN3A1 in EBV-infected individuals [129, 130]. In acute infectious mononucleosis, the expression of *γδ* TCR and the number of *γδ* T cells were increased analyzed by whole transcriptome profiling [27]. Overexpression of HSP60, HSP70, HSP90, and ULBPs, as protein ligands, can strengthen the recognition and effective cytotoxicity function of *γδ* T cells against virus-infected cells or malignant host cells [131, 132]. Human MutS homologue (including hMSH2/3/6), which is one kind of protein for DNA mismatch repair and also as a stress-induced protein ligand, is overexpressed in B lymphoblastic cells. This improves the recognition and effective cytotoxicity function of *γδ* T cells as well as protein ligands [133]. Besides, EBNA1 as nuclear antigen (also called latency I) is expressed on EBV-infected memory B cells and is indispensable for replication of viral genome. It can be recognized by V*γ*9V*δ*2 T cells and leads to V*γ*9V*δ*2 T cell expansion [128, 134]. Finally, activated V*γ*9V*δ*2 T cells could fight against EBV latency. In addition, activated V*γ*9V*δ*2 T cells which are based on FasL and TRAIL may exert effective elimination function of EBV-transformed lymphoblastoid cell lines [128]. Indeed, P-Ag-stimulated V*γ*9V*δ*2 T cells were able to prevent outgrowth of adoptively transferred EBV-transformed lymphoblastoid cell lines *in vivo* [135]. And adoptive transfer of V*γ*9V*δ*2 T cells could prevent tumorigenesis in mice in which EBV-associated lymphoma formation was induced by EBV infection [136]. In summary, V*γ*9V*δ*2 T cells combined with other cytotoxic innate lymphocyte subsets (NK T cells) can target various stages of EBV infection.

#### 3.2.4. Hepatitis B Virus (HBV) and Hepatitis C Virus (HCV)

HBV and HCV are involved in liver damage and can lead to viral hepatitis and even liver cancer [137, 138]. The liver is rich with multiple innate immune cells (like natural killer cells and *γδ* T cells) and plays an important role in innate immunity in the various stages of liver diseases [139–141]. Hepatic *γδ* T cells occupy a small proportion in total liver lymphocytes [139]. At the beginning, the number of V*δ*2 T cells, which account for a considerable proportion of *γδ* T cells in the liver, tends to decline accompanied by disease progression [142, 143]. Nevertheless, V*δ*1 T cells are expanded in liver diseases (especially acute-on-chronic liver failure infected by hepatitis B virus) when compared with V*δ*2 T cells and defense against liver damage by producing increased cytotoxicity and inflammatory cytokine [144]. Researchers recently revealed that the frequency of *γδ* T cell subsets (both V*δ*1 and V*δ*2) has increased in HBV-infected patients without symptoms. In HBV-infected patients, increased effector memory V*δ*2 T cells play a protective role by producing interferon-*γ* [145]. But in chronic HCV-infected patients, activation and differentiation of V*δ*2 T cells exert cytotoxicity via acquisition and expression of cytotoxic natural killer-like phenotype to eradicate the virus instead of producing interferon-*γ* [146]. Interestingly, *γδ* T cells could strengthen TNF-*α* production (induce IFN-*γ* expression) and CD107a expression (a functional marker for cytotoxicity) with antiviral drug interferon-*α* treatment. In other words, interferon-*α* can enhance cytotoxic function of *γδ* T cells in chronic HBV infection [147]. Moreover, peripheral V*δ*2 T cells activated by nonpeptidic antigens (such as pyrophosphomonoesters) can inhibit the replication of HCV via noncytolytic antiviral ability [148]. In contrast, it has been reported that in HBV-infected immunocompetent mice, *γδ* T cells mediated CD8^+^ T cell exhaustion by mobilizing myeloid-derived suppressor cell (MDSC) infiltration to the liver in HBV-induced tolerance [149].

### 3.3. Parasite

#### 3.3.1. Plasmodium

Malaria caused by *Plasmodium* occurs in tropical and subtropical regions and endangers the physical health. An earlier report demonstrated that conventional T cells (CD4^+^ and CD8^+^ T cells) exhibit a protective role in the elimination of *Plasmodium falciparum* [150]. Accumulating findings indicate that *γδ* T cells play a key role in defending against *Plasmodium* infection. *γδ* T cells are found increased during *Plasmodium* infection [151]. In *γδ* T cell depletion mice, the level of protective antibody (IgG2a) which eradicates the malaria parasite exhibits an apparent decline when compared with control [152]. In mouse models without sufficient *γδ* T cell, it was discovered that, in the context of agonistic anti-CD40 antibody, *γδ* T cells are involved in controlling *Plasmodium berghei* XAT (PbXAT). Afterwards, DCs can be activated by unconventional T cells by means of CD40 ligand expression, and whereafter, helper T lymphocyte 1 cells exert their effector response defending against *Plasmodium* via Th1 differentiation during PbXAT infection [152–154]. In addition, cytokines such as IL-12 and TNF are also crucial for controlling *Plasmodium* infection and decrease the risk of fever, clinical malaria, and parasitemia [155]. IL-12 and IL-18 are essential for expression of TIM3 (T cell immunoglobulin domain and mucin domain 3), one member of the TIM protein family, in *γδ* T cell, which could offer clinical malaria important opportunities for risk reduction [156]. Especially, IL-17A, which is largely produced by *γδ* T cells, could slow down the course of diverse pathogen infections. According to the report, IL-17A-producing *γδ* T cells in combination with monocytes are involved in the early process of fighting against parasites [157]. Some cytokines and chemokines (such as TNF and MIP-1*β*/1*α*) which increase the risks of severe malaria, however, are derived from *γδ* T cell [158]. Collectively, cytokines and chemokines have dual effects on *Plasmodium* infections.

Different subgroups of *γδ* T cell play various roles in controlling *Plasmodium* infections. V*γ*9V*δ*2T cells activated by *P. falciparum* antigens produce cytotoxic granules to kill merozoites and control parasite density during the blood stage of infection [159]. The proportion of V*δ*2^+^*γδ* T cells increased in previously naïve adults following malaria infection. But children with repeated malaria were associated with reduced percentages of V*δ*2^+^*γδ* T cells and cytokine secretion and increased expression of immunoregulatory genes. Taken together, the loss and dysfunction of V*δ*2^+^*γδ* T cells in children with repeated malaria may lead to clinical tolerance of the parasite [160]. Moreover, the diminished V*δ*2^+^*γδ* T cell proinflammatory cytokine production in this situation was associated with expression of the immunoregulatory markers TIM3 and CD57. Higher V*δ*2^+^*γδ* T cell proinflammatory cytokine production was associated with protection from subsequent *P. falciparum* infection [161]. Recently, it was discovered that both reduction and dysfunction of V*δ*2^+^*γδ* T cells promote the expression of CD16 which causes V*δ*2^+^*γδ* T cells to exhibit inefficient recognition of nonpeptidic antigens [162]. V*γ*1^+^*γδ* T cells are also important for defense against *Plasmodium* infection. During early *Plasmodium berghei* XAT (PbXAT) infection stage, expanding V*γ*1^+^*γδ* T cells promotes CD40 ligand expression and IFN-*γ* secretion. CD40 ligand- (CD40L-) CD40 signaling activates DCs to induce protective immunity. It was manifested that the V*γ*1^+^*γδ* T cell response is dependent on IFN-*γ*-activated DCs [163]. Nonetheless, at the late stage, the IFN-*γ* positivity of V*γ*1^+^*γδ* T cells is reduced due to *γδ* T cell dysfunction. Indeed, V*γ*1^+^*γδ* T cells promote inhibitory receptor expression, such as PD-1, LAG-3, and TIM3 at the late stage [163].

## 4. Possible *γδ* T Cell-Based Clinical Application

Bisphosphonates (also called aminobisphosphonates (ABP)) are commonly used to activate V*γ*9V*δ*2 T cells via accumulating and elevating the level of cellular IPP and its metabolites [164]. Pamidronate (PAM) and zoledronate (Zol) are bisphosphonates that can inhibit the IPP-metabolizing enzyme farnesyl diphosphate synthase (FDPS) which is a key enzyme of the mevalonate pathway [165, 166]. PAM is considered as an economical and practical way to activate V*γ*9V*δ*2 T cells [167]. In humanized mouse models, it is reported that PAM reduces disease severity and mortality and controls lung inflammation and viral replication after human influenza virus infection [168]. Zol is broadly exploited to enhance adoptive cancer immunotherapy and stimulate effector *γδ* T cells with antitumor activity [169, 170]. However, ABP as an anti-infection agent have certain limitations in clinical use. Intravenous infusion of ABP gives rise to immune-mediated diseases (such as persistent autoimmune syndromes) because of TNF-*α* and IFN-*γ* release by V*γ*9*δ*2 T cells which will induce inflammatory response or acute clinical response [171]. ABP affect oral absorption and inhibit bone resorption and even lead to bone side effects in cancer treatment [172]. Tetrakis-pivaloyloxymethyl 2-(thiazole-2-ylamino) ethylidene-1,1-bisphosphonate (PTA) as a synthetic bisphosphonate prodrug can also inhibit FDPS. It can get inside the cells where it is converted into acid enzymes with activity by intracellular esterases [173]. PTA could activate the expansion of peripheral blood V*γ*9*δ*2 T cells which are separated from cancer patients (prostate and breast cancer) [174]. Compared with Zol, PTA activates *γδ* T cell expansion more effectively and produces more cytokines (TNF-*α* and IFN-*γ*) [173].

Besides P-Ag-induced activation of *γδ* T cells, BTN3A-specific monoclonal antibody (mAb) 20.1 can also activate V*γ*9V*δ*2 TCR by CDR3 of V*γ*9 and V*δ*2 chain responsiveness to mAb 20.1 [175]. Meanwhile, mAb 20.1 can interfere with the P-Ags-response [175]. Thus, BTN3A-specific antibody may be useful agents against pathogen infections.

Adoptive transfer of *γδ* T cells by intravenous infusion is the most common way for the clinical trials of patients [176, 177]. Adoptive transfer therapy is confirmed as a safe way without requiring preconditioning to expand V*γ*9V*δ*2 T cells and has been reported in many studies [178, 179]. Researchers recently pay more attention to not only the safety but also the clinical effects of *in vitro* expanded *γδ* T cells in multiple ways including DNA copy number and negative conversion rate of HbeAg during active HBV infections (https://www.clinicaltrials.gov/). In nonhuman primate models infected by *Mycobacterium tuberculosis*, adoptive transfer of V*γ*9V*δ*2 T cells has no or reduced tuberculosis dissemination when compared with control [180]. V*γ*9V*δ*2 T cells by adoptive transfer therapy display central/effector memory and exert their effector function defense against MTB infections via secreting anti-*M. tuberculosis* cytokines and inhibiting intracellular bacteria [180]. Adoptive transfer therapy based on *γδ* T cells is also applicable for treatment of a range of cancers including renal cancer, breast and cervical cancer, and non-small-cell lung cancer [181, 182]. Interestingly, it is more vulnerable to accomplish successfully adoptive transfer of *γδ* T cells following ABP treatment [183].

An earlier study reports that low-dose IL-2 could synergize with nitrogen-containing bisphosphonate or pyrophosphomonoester drugs to expand V*γ*9V*δ*2 T cells [71]. Phosphoantigens combined with IL-2 are an efficient method to activate and expand V*γ*9V*δ*2 T cells both *in vitro* and *in vivo* [74, 184]. Expression of NO synthase (NOS2) exerts profound influence on *γδ* T cell properties, including IL-2 secretion, its expansion, and glycolysis metabolism. Recently, there is a report that IL-2 is not completely necessary for V*γ*9V*δ*2 T cells in adoptive immunotherapy [174]. IL-18 represents a new potential treatment for HIV-positive individuals since it activates V*γ*9V*δ*2 T cell responses to phosphoantigen [185].

Broadly speaking, *γδ* T cell-based clinical application has both advantages and limits in controlling and even eliminating pathogen infections. *γδ* T cells have the following extraordinary advantages: firstly, *γδ* T cell-based clinical application emphasizes the importance of host immune response instead of pathogens themselves. Secondly, *γδ* T cells rapidly gather at the site of infection and exert effective function of elimination of pathogens. Thirdly, *γδ* T cells play multiple roles in controlling infection on the basis of different subsets of *γδ* T cells with different functions and *γδ* T cells act as functionally diversified cells such as APC and potential regulatory T cells. Fourthly, though *γδ* T cells make up a very small proportion of T cells in the human body, they can be directly activated by phosphoantigens or indirectly activated by drugs that induce IPP accumulation or monoclonal antibody, both of which are economical and practical. Fifthly, there is a relatively safe way for the clinical trials of patients: adoptive transfer of *γδ* T cells by intravenous infusion. However, current application of conventional therapy also has certain limitations in clinical use. It has been reported that phosphoantigen reapplication may lead effector cells to an incapable, exhausted, and even dead condition [186]. Irrational drug use like overdoses may lead to autoimmune diseases. Moreover, activated *γδ* T cells by drugs like ABP release many proinflammatory cytokines and may also give rise to immune-mediated diseases such as persistent autoimmune syndromes. Therefore, it is important to confirm both the safety and the dose of clinical medication in the future and *γδ* T cell-based immune therapy still needs further discussion and research.

Above all, though the mentioned potential therapeutic methods have some limitations, it put forward ideas and methods for further clinical research. To achieve an effective and safe treatment on infected patients, no doubt, we need a broader and deeper understanding of effector function of different subgroups of human *γδ* T cells.

## 5. Summary

Since the diverse subpopulations of *γδ* T cells possess different biological characteristics, they play different roles in various infectious illnesses induced by bacteria, viruses, and parasites. Different kinds of antigens associated with various pathogen infections including nonpeptidic antigens (P-Ags), MHC molecules, and lipids could be directly or indirectly recognized by *γδ* T cells. Some *γδ* T cells are immediately activated, while some *γδ* T cells also need a second signal costimulation. The activation and expansion of *γδ* T cells exert their effector function during pathogen infections. Growing evidence suggests that *γδ* T cells act as a link to connection innate with adaptive immunity. It is intriguing to find that *γδ* T cells can also work as APC to present pathogen infection-associated antigen to CD4^+^ and CD8^+^ T cells. In addition, *γδ* T cells exert their protective function in the elimination of pathogens and tissue repair via producing cytokines, chemokines, and lytic enzymes and cytotoxic and noncytolytic antiviral activities. *γδ* T cells can also promote DC maturation and provide B cell help to produce antibody. Collectively, *γδ* T cells play a significant role in the elimination of pathogens. In view of the promising implications of *γδ* T cells to treat infectious diseases in preclinical studies, it is hoped that *γδ* T cells will provide a potentially effective new way to treat infectious diseases.
